# RIOK2 is negatively regulated by miR‐4744 and promotes glioma cell migration/invasion through epithelial‐mesenchymal transition

**DOI:** 10.1111/jcmm.15107

**Published:** 2020-03-03

**Authors:** Yunnong Song, Cheng Li, Lei Jin, Jingsong Xing, Zhuang Sha, Tong Zhang, Daofei Ji, Rutong Yu, Shangfeng Gao

**Affiliations:** ^1^ Institute of Nervous System Diseases Xuzhou Medical University Xuzhou China; ^2^ Department of Neurosurgery The Affiliated Hospital of Xuzhou Medical University Xuzhou China

**Keywords:** fibronectin, glioma, miR‐4744, N‐cadherin, RIOK2, β‐catenin

## Abstract

RIOK2 is a member of RIO (right open reading frame) kinase family. Recent studies have revealed the involvement of RIO kinases in glioma cell growth and expansion. However, the role and mechanism of RIOK2 in glioma cell migration and invasion remain unclear. Wound healing assay, Transwell assay and real‐time quantitative PCR (qRT‐PCR) detection of matrix metalloproteinases (MMPs) were used to evaluate the migration/invasion of glioma cells. Western blot and qRT‐PCR were employed to measure the expression of epithelial‐mesenchymal transition (EMT) markers. Dual luciferase reporter assay was performed to determine the binding between RIOK2 and miR‐4744. In addition, RIOK2 and miR‐4744 levels were quantified by qRT‐PCR and/or immunohistochemistry in glioma tissues. Transfection of RIOK2 siRNAs significantly inhibited glioma cell migration and invasion and down‐regulated the expression of MMPs (MMP2 and MMP9) and mesenchymal markers (N‐cadherin, β‐catenin, Twist1, fibronectin, ZEB‐1) in glioma cells. Overexpression of RIOK2 showed the opposite effects. MiR‐4744 directly bound to the 3'‐untranslated region of RIOK2 and negatively regulated the expression of RIOK2. Up‐regulation of miR‐4744 inhibited the migration and invasion of glioma cells. Overexpression of RIOK2 could reverse the effects of miR‐4744 up‐regulation on the migration, invasion and EMT process in glioma cells. Moreover, RIOK2 was high, while miR‐4744 was low in glioma tissues, and a negative correlation was found between them. These results suggest that RIOK2 is post‐transcriptionally targeted by miR‐4744, the low miR‐4744 and high RIOK2 levels in glioma may contribute to tumour cell infiltration through promoting the EMT.

## 1 INTRODUCTION

Glioma is the most common intracranial primary tumour, with high malignancy, rapid progression and poor prognosis.^1^ Although surgery remains the mainstay for the treatment of gliomas, high‐grade gliomas exhibit a highly invasive clinical behaviour that, in most cases, precludes complete surgical resection.^2^ Therefore, searching novel therapeutic targets to combat glioma growth and infiltration is critical for the treatment of this currently incurable type of cancer.

RIOK2 is a member of the RIO (right open reading frame) family that also includes RIOK1 and RIOK3.^3^ RIO family proteins have the properties of a kinase, but they have no domain similar to a typical kinase, so they are called atypical kinases.^4^ A growing number of studies have shown that RIO kinase is involved in pre‐rRNA processing and ribosomal biosynthesis.^5^ Many proteins associated with ribosome biogenesis play important parts in cell cycle progression.^6^ Knockdown of RIOK1 arrests yeast cells at S and mitosis phases.^7^ RIOK2 acts as the substrate of PLK1 and is required for the proper mitotic progression in Hela cells.^8^ In addition, our recent work has found that down‐regulation of RIOK3 causes G1 arrest, whereas overexpression of RIOK3 accelerates cell cycle progression in glioma cells.^9^ Overall, RIO kinases play a key role in both ribosome synthesis and cell cycle progression, indicating their potential roles in tumour cell growth and expansion.

In recent years, studies on the involvements of RIO kinases in tumours have gradually increased. Knockdown of RIOK1 inhibits the proliferation, migration and invasion in breast, lung and colon cancer cells.^10^ Stable overexpression of RIOK3 promotes proliferation, invasion and migration of glioma cells, while silencing of RIOK3 inhibits proliferation, migration and invasion, and induces apoptosis in glioma cells.^9^ In addition, RIOK2 and RIOK1 are highly expressed in glioblastoma cells, overexpression of RIOK1/2 promotes cell proliferation, and down‐regulation of RIOK1/2 causes apoptosis and increased sensitivity to chemotherapy.^11^ Thus, RIO kinases may play a promoting role in the progression of malignant tumours, but it should be noted that the role of RIOK2 in cancer cell migration and invasion has not been elucidated yet.

MicroRNA (miRNA) is a kind of endogenous small RNA with a mature form of approximately 18‐22 nucleotides. It is considered that miRNAs can regulate more than 70% of human genes by binding to the 3'‐untranslated region (3'‐UTR) of other RNAs.^12^, ^13^ In recent years, studies have found that miRNAs affect biological behaviours such as tumour occurrence, proliferation and invasion mostly by regulating target genes.^14^ MiRNAs act as oncogenes or tumour suppressors, depending on their target genes.^15^, ^16^, ^17^ Extensive studies have revealed the dysregulation of miRNAs in the progression of gliomas. For example, miR‐210 strictly regulates the expression of NeuroD2, and overexpression of NeuroD2 reduces the invasiveness of glioblastoma under hypoxia conditions.^18^ The level of miRNAs in cerebrospinal fluid and brain tissue of glioma patients is a reliable marker and may have diagnostic value for glioma.^19^ By searching TargetScan, miRDB and microT‐CDS, we found that miR‐4744 may be the upstream miRNA regulating RIOK2. MiR‐4744 is a new miRNA identified by second‐generation sequencing in breast cancer tissues, and its expression is increased in breast cancer tissues.^20^

Glioblastoma cells are highly invasive because of the high migration potential and the ability to invade surrounding tissues. Epithelial‐mesenchymal transition (EMT) is one of the mechanisms leading to glioblastoma invasion characteristics.^21^ EMT is a biological process that undergoes a variety of biochemical changes from polarized epithelial cells to mesenchymal phenotype, which is characterized by loss of epithelial markers (eg E‐cadherin) and gain of mesenchymal markers (eg N‐cadherin, β‐catenin, Twist1). As a result, tumour cells with mesenchymal phenotype lose their apical basolateral polarization and acquire fibroblast‐like morphology, increasing their ability to spread to surrounding tissues or to distant locations.^22^

In this study, we first studied the role of RIOK2 in glioma cell migration, invasion and EMT process using small interfering RNA (siRNA) and overexpression lentivirus of RIOK2. Then, we searched the databases such as TargetScan, miRDB and microT‐CDS for predicting the possible miRNAs that regulate RIOK2 post‐transcriptionally. We confirmed that miR‐4744 directly bound to the 3'‐UTR of RIOK2 and negatively regulated RIOK2 expression in glioma cells. Furthermore, we found that miR‐4744 inhibited the migration, invasion and EMT process in glioma cells, and overexpression of RIOK2 partially reversed the suppressed effects of miR‐4744 on cell migration, invasion and EMT. Finally, RIOK2 expression was found to up‐regulate at both mRNA and protein levels, while miR‐4744 level was down‐regulated in glioma tissues, and a negative correlation was found between them.

## 2 MATERIALS AND METHODS

### 2.1 Patients and samples

All glioma tissue specimens (obtained during surgical resection) and nontumour brain tissue specimens (obtained by patients undergoing decompression surgery for traumatic brain injury) were collected from the Affiliated Hospital of Xuzhou Medical University. No patient has received chemotherapy, immunotherapy or radiotherapy. For quantitative Real‐Time PCR (qRT‐PCR) analysis, we stored fresh specimens at −135°C immediately after surgical resection; the clinicopathological data of these subjects were given in Table S1. For immunohistochemical analysis, the specimens were fixed in 10% buffered formalin and embedded in paraffin for sectioning; the clinicopathological information of these subjects was available in the Table S1 of our previously published paper.^9^ All glioma specimens had a confirmed pathological diagnosis and were classified according to the criteria of the World Health Organization (WHO). All subjects signed informed consent forms, and the Ethics Committee of Xuzhou Medical University approved the study.

### 2.2 Cell line and cell culture

The HEK293T cells and the human glioma cell lines U251, U87 and U118 were purchased from the Shanghai Cell Bank, Type Culture Collection Committee, Chinese Academy of Sciences. The identities of U251, U87 and U118 cell lines were confirmed by DNA profiling test (STR). The cells were grown in DMEM (293T, U118 and U251) or MEM (U87) supplemented with 10% foetal bovine serum (Gibco). All cell lines were cultured in a cell incubator with a 5% CO_2_ atmosphere under saturated humidity at 37°C.

### 2.3 RNA extraction and qRT‐PCR

Total RNA was extracted from tissues or cells using TRIzol (Invitrogen, Waltham, MA). To detect miR‐4744, we employed a cDNA reverse transcription kit (Takara, Dalian, China) and a pair of miR‐4744‐specific reverse transcription primer (Biomics Biotech, Nantong, China) to perform reverse transcription, and then used a SYBR Green PCR Master kit (Takara) and a miR‐4744 qRT‐PCR Detection Primer Set (Biomics Biotech) to perform qRT‐PCR. To detect U6 snRNA and other genes, reverse transcription and qRT‐PCR were performed using the similar protocol as described elsewhere.^9^ The forward and reverse primers were shown in Table S2. Data were automatically collected and processed using the Applied Biosystems 7500. The absolute amount of target genes and miR‐4744 were calculated and normalized by that of β‐actin and U6, respectively, according to our previous method.^23^

### 2.4 Protein extraction and Western blot

Total protein was extracted from tissues or cells, and a BCA Protein Assay Kit (Beyotime, Haimen, China) was used to determine protein concentration. Equal amounts of total protein were loaded for Western blot analysis as described in our recent paper.^9^ The primary antibodies used were RIOK2 (1:500, Novus Biologicals), N‐cadherin (1:1000, Abcam), β‐catenin (1:2000, Cell Signaling Technology), Twist1 (1:500, Santa Cruz Bio), fibronectin (1:1000, BD), ZEB‐1 (1:500, Proteintech) and β‐actin (1:1500, Santa Cruz Bio). Band densities were quantified using Image J software (National Institutes of Health, Bethesda, MD). Relative protein levels were determined by normalizing the densitometry value of the proteins of interest to that of β‐actin.

### 2.5 Transfection

#### 2.5.1 MiRNA/siRNA transfection

RIOK2 siRNAs, miR‐4744 mimics and the negative controls (Table S2) were synthesized by Biomics Biotech. The cells were grown to 60%‐70% confluence in six‐well plates, then transfected with the purchased oligonucleotides using Lipofectamine 2000 (Invitrogen) according to the manufacturer's instructions.

#### 2.5.2 Plasmid transfection

Cells were seeded on 6‐cm dishes. After the cells reached a confluence of 70%‐80%, the medium was removed and replaced with 1 mL fresh medium for half an hour. Concomitantly, 1 μg Myc‐RIOK2 plasmid^8^ and 3 μL PolyJet (SignaGen, Gaithersburg, MD) were added into 50 μL DMEM medium respectively and then mixed thoroughly. The latter was added into the former and then placed at room temperature for 10 to 15 minutes. The mixture was then evenly added into the dish, which was subsequently placed in the incubator. Six hours later, we replaced the medium with 2 mL fresh medium and continued to culture the cells for subsequent experiments.

### 2.6 Lentivirus construction, production and infection

The human RIOK2 (Accession number: NM_018343) was inserted into the pCDH‐GFP‐puro vector plasmid at Nhe I and BamH I sites. The lentiviruses were produced in HEK293T cells and used to infect glioma cells according to our previously reported protocol.^9^ Forty‐eight hours after infection, the virus‐infected cells were cultured in the medium containing 2.5 μg/mL puromycin for selection. The surviving cells were used in the subsequent experiments. In order to distinguish it from Myc‐RIOK2 plasmid, the lentivirus plasmid of RIOK2 here was named ‘RIOK2’, and the corresponding control was named ‘Vector’.

### 2.7 Cell viability detection

Cell viability was measured using a Cell Counting Kit‐8 (CCK‐8, Dojindo, Japan) as described previously.^24^, ^25^ Cell viabilities at individual time‐point were normalized to those at 6 hours for each group.

### 2.8 Wound healing assay

We used wound healing assay to evaluate cell migration behaviour in glioma cells. A plastic pipette tip was used to scratch the monolayer cells, then the dead cells were washed out of the plate and serum‐free medium was added. Images of three randomly at the lesion border were captured under an Olympus IX‐71 inverted microscope at the designated times (0 hours, 24 hours and 48 hours). The wound healing rate was calculated based on the captured images.

### 2.9 Transwell migration and invasion assays

A Transwell system with a polycarbonate filter membrane was used to perform cell migration and invasion assays as described in the literature.^26^, ^27^ Briefly, cells were seeded at a density of 1 × 10^4^ cells in 200 µL of serum‐free medium in the upper chamber. The bottom chamber was filled with 500 µL of culture medium containing 10% FBS, then incubated at 37°C for 24 hours. To assess invasion ability, we used 10 μg of Matrigel (BD) to pre‐coat the filters. Three fields of adherent cells in each well were randomly captured under an Olympus IX71 inverted microscope. The migrating and invading cells were counted on the captured images.

### 2.10 Immunofluorescence

Immunofluorescence was performed according to a previously described protocol.^28^ The primary antibodies against N‐cadherin (Abcam) and β‐catenin (Cell Signaling Technology) were added at 1:200 and 1:100, respectively. The secondary antibody conjugated to Alexa Fluor 488 (1:400, Invitrogen) or to Alexa Fluor 594 (1:200, Invitrogen) was employed to visualize the primary antibody. Cell nuclei were stained with 4,6‐diamidino‐2‐phenylindole (DAPI; 1:1000; Sigma). The cells were observed and recorded using a Leica fluorescence microscope (Germany).

### 2.11 Dual luciferase gene reporter assay

MiRDB online prediction software (http://www.mirdb.org/) was chosen to predict the binding site of miR‐4744 at the 3'‐UTR of RIOK2. The predicted binding site of RIOK2 was inserted into the psiCHECK2 plasmid to construct wild‐type‐RIOK2‐3'‐UTR plasmid. Meanwhile, mutant‐RIOK2‐3'‐UTR plasmid was constructed using the same plasmid and used for negative control since it could not bind to miR‐4744 any longer. Both plasmids were obtained from Bio‐Transduction Lab Co. Ltd (Wuhan, China). When cells reached 80%‐90% confluence, we set the following groups and performed the corresponding transfections: wild‐type‐RIOK2‐3'‐UTR + miR‐NC group, wild‐type‐RIOK2‐3'‐UTR + miR‐4744mimics group, mutant‐RIOK2‐3'‐UTR + miR‐NC group, mutant‐RIOK2‐3'‐UTR + miR‐4744mimics group. Luciferase Reporter Assay System was used to measure the luciferase activity according to the manufacturer's instructions (Promega).

### 2.12 Immunohistochemistry and cell counting

RIOK2 immunoreactivity (IR) was detected by immunohistochemistry and quantified by cell counting, as described in our previous paper.^9^ The RIOK2 antibody (Novus Biologicals) was used at 1:50.

### 2.13 Statistical analysis

In vitro experiments were repeated at least three times, and the data were expressed as the means ± SD Comparisons between two groups were performed using Student's *t* test, and differences among three groups were determined using one‐way analysis of variance (ANOVA) followed by Dunnett's or Tukey *post hoc* test. Differences between the nontumour group and the glioma subgroups were evaluated using the Kruskal‐Wallis test and the Mann‐Whitney U test. Correlations were analysed by the Spearman correlation test. Statistical analyses were performed using SPSS version 19.0 (SPSS Inc, Chicago, IL). Tests were two‐tailed, and *P* < .05 was considered statistically significant.

## 3 RESULTS

### 3.1 Down‐regulation of RIOK2 inhibits glioma cell migration

**Figure 1:**
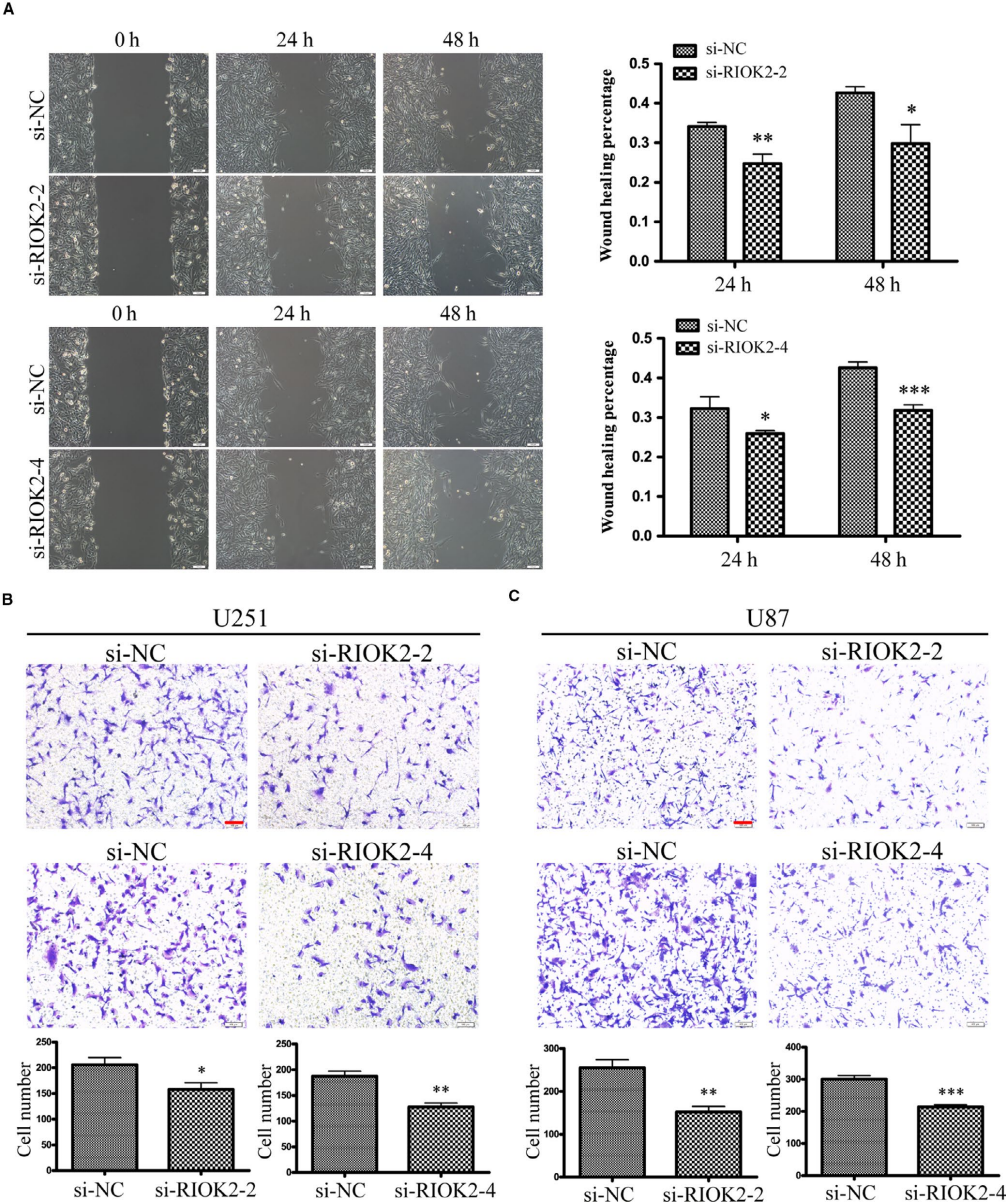
Down‐regulation of RIOK2 inhibits glioma cell migration. (A) Wound healing assay was used to assess the effects of RIOK2 down‐regulation on cell migration at 24 h and 48 h in U251 cells. Representative images were shown on the left column, and quantitative analyses of the wound healing rate were shown on the right column. (B‐C) Transwell assay was performed to evaluate the effects of RIOK2 down‐regulation on cell migration in U251 and U87 cells. Representative images were shown in the upper panel and quantitative analyses of the number of cells migrating to the chamber were shown in the lower panel. Scale bars: 100 μm. *P < .05; **P < .01; ***P < .001

The expression of RIOK2 was down‐regulated in glioma cells by transfection of RIOK2 siRNAs (si‐RIOK2‐2 and si‐RIOK2‐4). Western blot analysis showed that RIOK2 was successfully down‐regulated by both siRNAs in U251 and U87 cells (Figure S1). Since RIOK2 was reported to inhibit glioma cell proliferation,^11^ we firstly used CCK8 assay to measure cell viability. It was found that silencing of RIOK2 resulted in a significant decrease in the cell viability at 72 hours for U251 cells and at 48‐72 hours for U87 cells (Figure S3). Next, we used wound healing and Transwell assays to assess the effects of down‐regulation of RIOK2 on glioma cell migration. Wound healing assay displayed that knockdown of RIOK2 led to a significant decrease in the wound healing rate at 24 hours (si‐RIOK2‐2: *P* = .003, si‐RIOK2‐4: *P* = .023) and 48 hours (si‐RIOK2‐2: *P* = .011, si‐RIOK2‐4: *P* < .001) in U251 cells (Figure 1A). Transwell migration assay showed that the number of U251 cells (si‐RIOK2‐2: *P* = .012, si‐RIOK2‐4: *P* = .001) and U87 cells (si‐RIOK2‐2: *P* = .002, si‐RIOK2‐4: *P* < .001) migrating to the chamber was significantly decreased after RIOK2 was down‐regulated (Figure 1B‐C). These results suggested that down‐regulation of RIOK2 inhibited glioma cell migration.

### 3.2 Down‐regulation of RIOK2 inhibits glioma cell invasion

**Figure 2:**
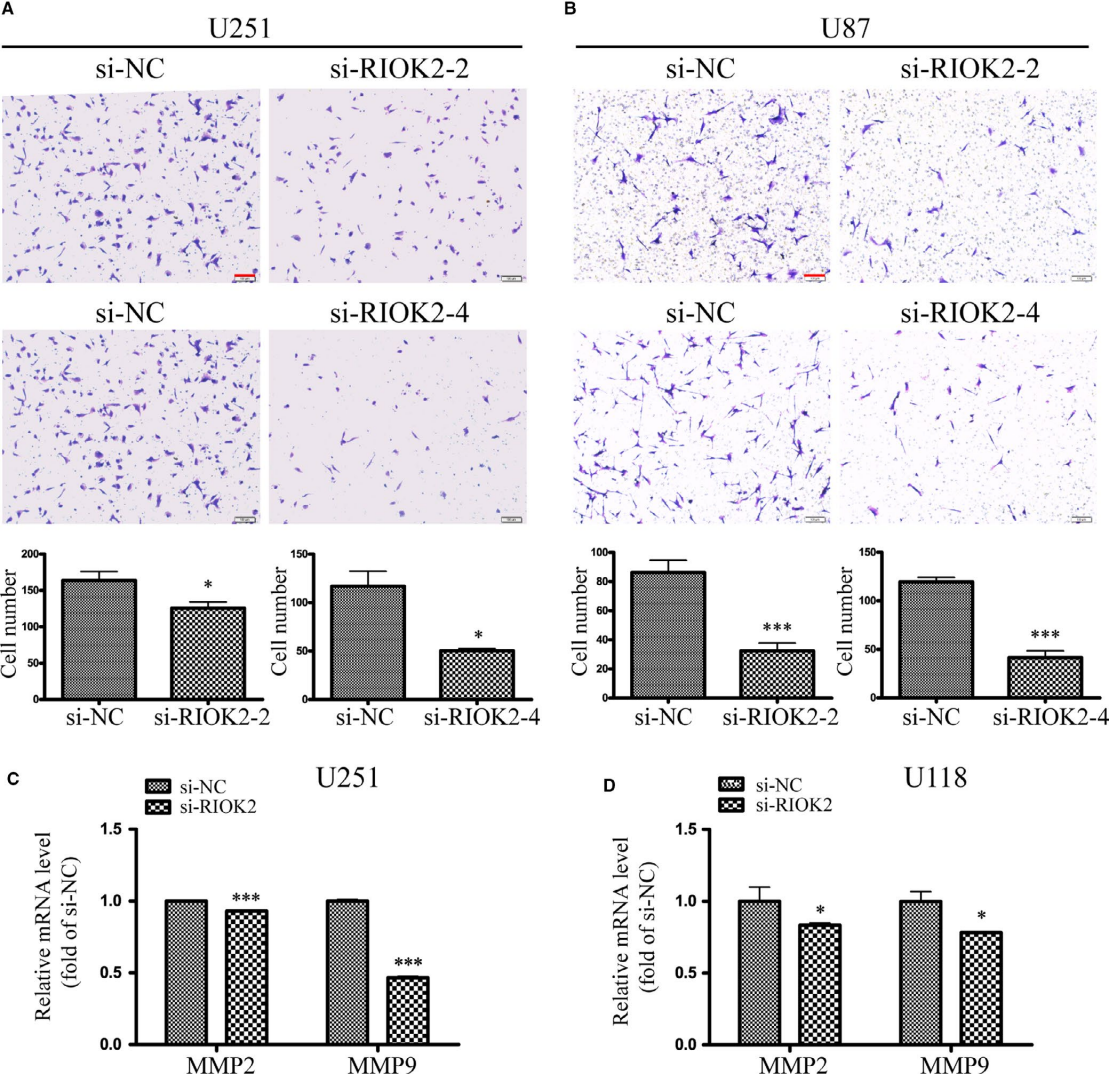
Down‐regulation of RIOK2 inhibits glioma cell invasion. (A‐B) Transwell assay was employed to assess the effects of RIOK2 down‐regulation on cell invasion in U251 (A) and U87 (B) cells. Representative images were shown in the upper panel, and quantitative analyses of the number of cells passing through the Matrigel were shown in the lower panel. (C‐D) QRT‐PCR was used to measure the changes of MMP2 and MMP9 in U251 (C) and U118 (D) cells following RIOK2 knockdown. Scale bars: 100 μm. *P < .05; ***P < .001

Transwell invasion assay and qRT‐PCR detection of matrix metalloproteinases (MMPs) were used to evaluate the effects of down‐regulation of RIOK2 on glioma cell invasion. Transwell invasion assay showed that the number of cells passing through the Matrigel was significantly reduced after RIOK2 was down‐regulated in U251 cells (si‐RIOK2‐2: *P* = .011, si‐RIOK2‐4: *P* = .016, Figure 2A) and U87 cells (si‐RIOK2‐2: *P* = .001, si‐RIOK2‐4: *P* < .001, Figure 2B). QRT‐PCR showed that the mRNA levels of MMP2 and MMP9 were significantly decreased in U251 cells (all *P* < .001, Figure 2C) and U118 cells (all *P* = .046, Figure 2D). The above results indicated that down‐regulation of RIOK2 inhibited glioma cell invasion.

### 3.3 Down‐regulation of RIOK2 inhibits the EMT process in glioma cells

**Figure 3:**
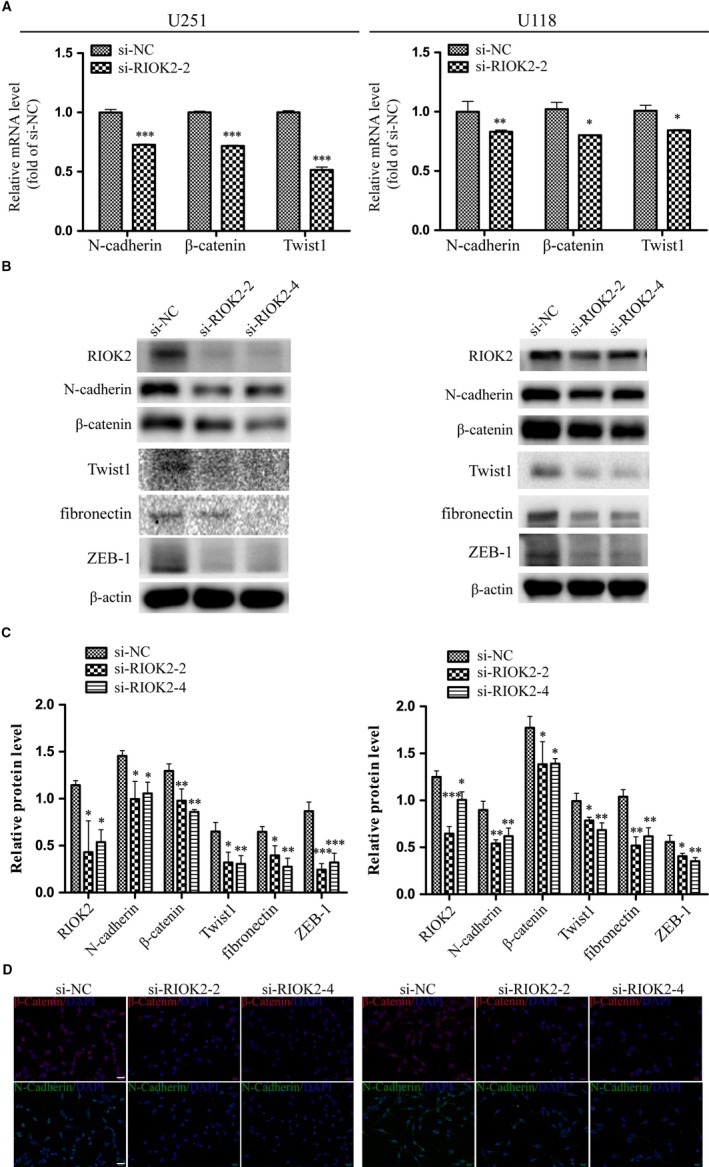
Down‐regulation of RIOK2 inhibits the EMT process in glioma cells. (A) QRT‐PCR was employed to measure the changes of EMT markers (N‐cadherin, β‐catenin and Twist1) in U251 and U118 cells following RIOK2 down‐regulation. (B‐C) Western blot was used to measure the changes of EMT markers (N‐cadherin, β‐catenin, Twist1, fibronectin and ZEB‐1) in U251 and U118 cells following RIOK2 knockdown. Representative blot images were shown in (B). Quantification graphs were shown in (C). (D) N‐cadherin and β‐catenin were observed by immunofluorescence after transfection of RIOK2 siRNAs in U251 and U118 cells. Scale bar: 50 μm; *P < .05; **P < .01; ***P < .001

The epithelial‐to‐mesenchymal transition (EMT) has been considered to be a key regulator of glioma cell invasiveness.^29^ QRT‐PCR and Western blot analyses were employed to detect the expression of important signalling molecules that mediate the EMT process in glioma cells. It was found that the mRNA levels of mesenchymal markers were significantly reduced in U251 cells (all *P* < .001) and U118 cells (N‐cadherin: *P* = .003, β‐catenin: *P* = .023, Twist1: *P* = .024) following RIOK2 down‐regulation (Figure 3A). Western blot analysis showed that transfection of RIOK2 siRNAs caused significant decreases in the protein levels of mesenchymal markers in U251 cells (N‐cadherin: *P* ≤ .018, β‐catenin: *P* ≤ .007, Twist1: *P* ≤ .011, fibronectin: *P* ≤ .021, ZEB‐1: *P* < .001) and U118 cells (N‐cadherin: *P* ≤ .008, β‐catenin: *P* ≤ .046, Twist1: *P* ≤ .017, fibronectin: *P* ≤ .002, ZEB‐1: *P* ≤ .017) (Figure 3B‐C). In addition, immunofluorescence staining further exhibited that knockdown of RIOK2 reduced the immunoreactivity of N‐cadherin and β‐catenin (Figure 3D). These data suggested that the down‐regulation of RIOK2 suppressed the mesenchymal phenotype of glioma cells.

### 3.4 Overexpression of RIOK2 promotes glioma cell migration and invasion

**Figure 4:**
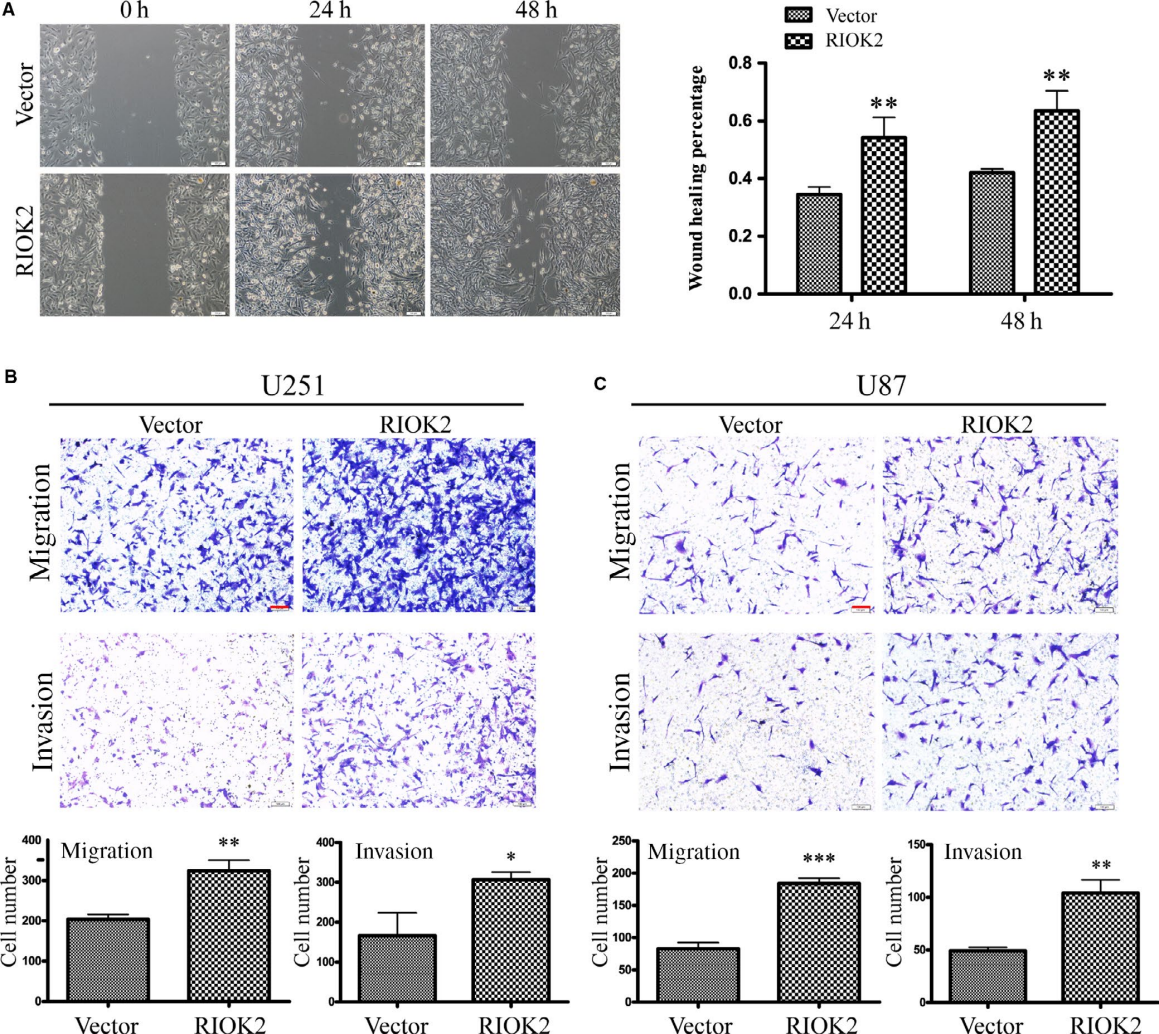
Overexpression of RIOK2 promotes glioma cell migration and invasion. (A) Wound healing assay was used to assess the effects of RIOK2 overexpression on cell migration at 24 h and 48 h in U251 cells. Representative images were shown on the left column, and quantitative analyses of the wound healing rate were shown on the right column. (B‐C) Transwell assay was performed to evaluate the effects of RIOK2 overexpression on cell migration and invasion in U251 and U87 cells. Representative images were shown in the upper panel, and quantitative analyses of the number of cells migrating to the chamber and passing through the Matrigel were shown in the lower panel. Scale bar: 100 μm. *P < .05; **P < .01; ***P < .001

RIOK2 was forced to expression in glioma cells by lentivirus‐mediated infection. Combined the bright field (BF) and GFP fluorescence, above 90% of U251 and U87 cells were infected. Western blot analysis displayed that exogenous RIOK2 was abundantly expressed in U251 and U87 cells (Figure S2). CCK8 assay showed that overexpression of RIOK2 caused a significant increase in the cell viability at 72 hours for U251 cells and at 48‐72 hours for U87 cells (Figure S3). We next used wound healing and Transwell migration assays to assess the influence of overexpression of RIOK2 on the migration of glioma cells. Wound healing assay showed that the wound healing rate of U251 cells in RIOK2‐overexpressing group was faster at 24 hours (*P* = .010) and 48 hours (*P* = .007) than that in the vector group (Figure 4A). Transwell migration assay showed that the number of U251 (*P* = .002) and U87 (*P* < .001) cells migrating to the chamber was significantly increased in the RIOK2‐overexpressing cells (Figure 4B‐C). Transwell invasion assay showed that overexpression of RIOK2 led to a significant increase in the number of cells crossing the Matrigel in U251 (*P* = .016) and U87 (*P* = .002) cells (Figure 4B‐C). These findings suggested that overexpression of RIOK2 promoted glioma cell migration and invasion.

### 3.5 MiR‐4744 directly binds to 3'‐UTR of RIOK2 and negatively regulates the expression of RIOK2

**Figure 5:**
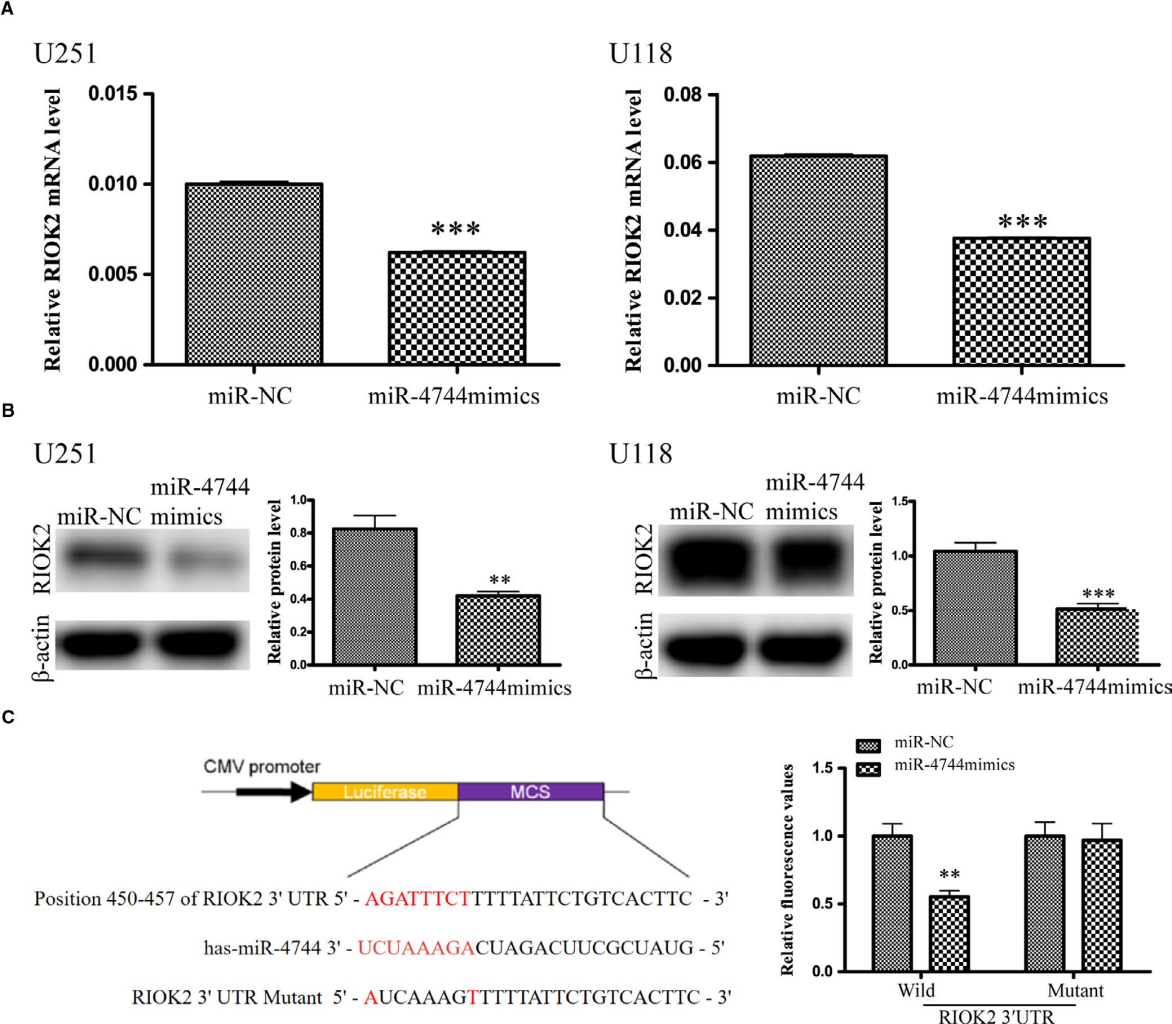
MiR‐4744 directly binds to 3'‐UTR of RIOK2 and negatively regulates the expression of RIOK2. (A‐B) QRT‐PCR and Western blot analyses were employed to evaluate the effects of miR‐4744 mimics treatments on the expression of RIOK2 in U251 and U118 cells. (C) Dual luciferase gene reporting assay was performed to determine whether miR‐4744 could bind to the 3’‐UTR of RIOK2. A scheme for the construction of wild‐type/mutant RIOK2 3'‐UTR plasmids was shown. Overexpression of miR‐4744 caused a significant decrease in the relative fluorescence value in the wild‐type RIOK2 3'‐UTR group, while it showed no significant influence on the relative fluorescence value in the mutant RIOK2 3'‐UTR group. **P < .01; ***P < .001

MicroRNAs post‐transcriptionally regulate other RNAs through binding to the 3'‐UTR of their targets. We searched databases such as TargetScan, miRDB and microT‐CDS to find the miRNAs that possibly bind to the 3'‐UTR of RIOK2. MiR‐4744 was found in all these databases. Next, we demonstrated that up‐regulation of miR‐4744 by its mimics significantly decreased the expression of RIOK2 at both mRNA (U251: *P* < .001; U118: *P* < .001) and protein (U251: *P* = .003; U118: *P* < .001) levels in glioma cells (Figure 5A‐B), indicating that RIOK2 is probably a target gene of miR‐4744. In order to prove that miR‐4744 directly binds to the 3'‐UTR of RIOK2, we constructed wild‐type and mutant 3'‐UTR of RIOK2 into dual luciferase plasmids. MiR‐4744 mimics treatments significantly decreased the luciferase activity in the wild‐type‐RIOK2‐3'‐UTR group (*P* = .002), whereas it showed no significant effects in the mutant‐RIOK2‐3'‐UTR group (Figure 5C). Therefore, we demonstrated that miR‐4744 directly bound to the 3'‐UTR of RIOK2 and could negatively regulate the expression of RIOK2.

### 3.6 Overexpression of RIOK2 could reverse the effects of miR‐4744 on glioma cell migration

**Figure 6:**
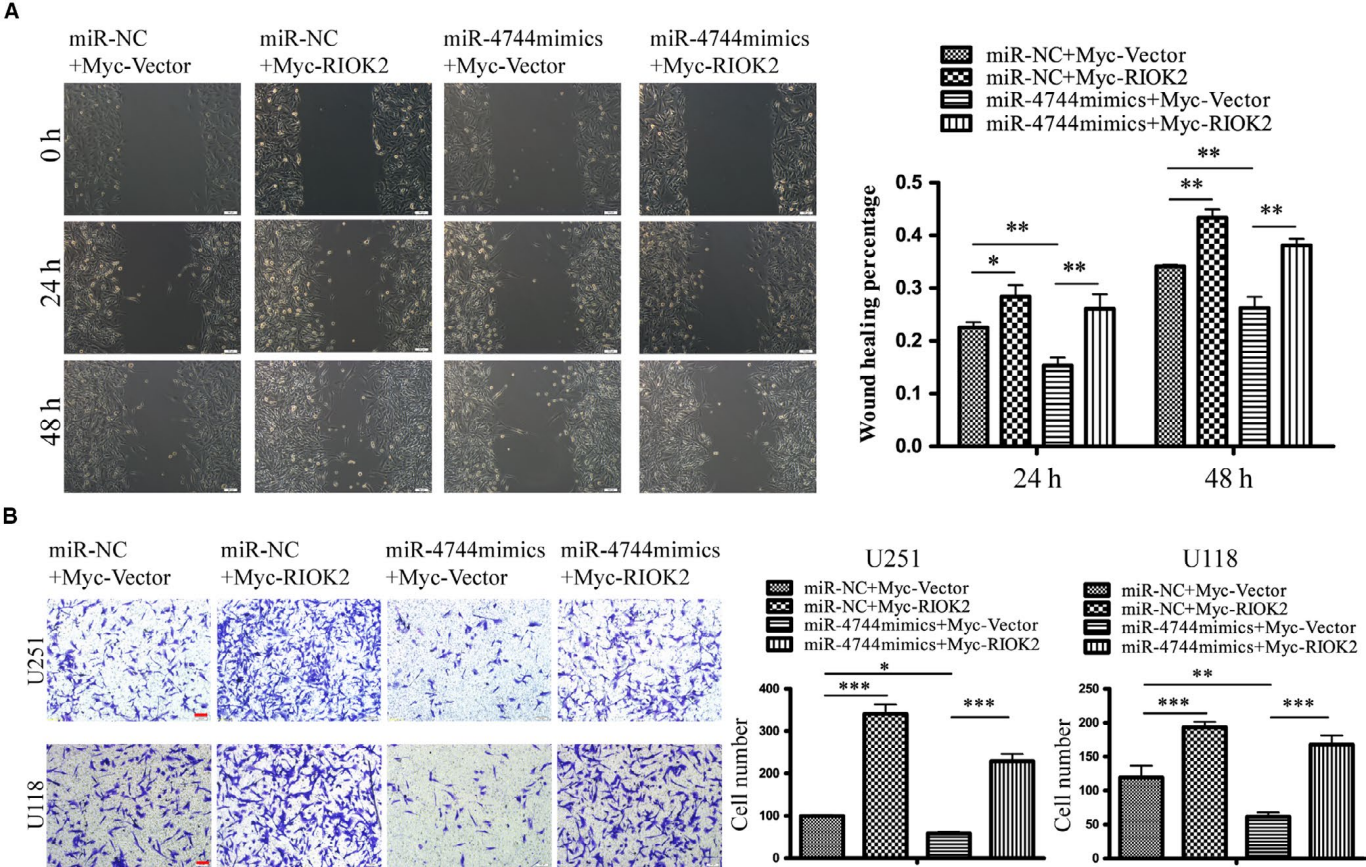
Overexpression of RIOK2 could reverse the effects of miR‐4744 on glioma cell migration. (A) Wound healing assay was performed to assess the reversal effects of RIOK2 overexpression on the decreased cell migration caused by up‐regulation of miR‐4744 at 24 h and 48 h in U251 cells. Representative images were shown on the left column, and quantitative analyses of the wound healing rate were shown on the right column. (B) Transwell migration assay was performed to assess the reversal effects of RIOK2 overexpression on the decreased cell migration caused by up‐regulation of miR‐4744 in U251 and U118 cells. Representative images were shown on the left column, and quantitative analyses of the number of cells migrating to the chamber were shown on the right column. Scale bar: 100 μm. *P < .05; **P < .01; ***P < .001

In order to verify whether miR‐4744 affects glioma cell migration and invasion through regulating RIOK2, we performed the following rescue experiments. Wound healing assay showed that overexpression of RIOK2 significantly increased the wound healing rate in U251 cells (24 hours: *P* = .026; 48 hours: *P* = .002, Figure 6A), which was in line with our results in Figure 4A. Up‐regulation of miR‐4744 led to a significant decrease in the wound healing rate compared with the miR‐NC group (24 hours: *P* = .009; 48 hours: *P* = .004, Figure 6A). More importantly, overexpression of RIOK2 partially reversed the decrease in the wound healing rate caused by up‐regulation of miR‐4744 (24 hours: *P* = .001; 48 hours: *P* = .003, Figure 6A). In addition, Transwell migration assay further confirmed the reversal effects of overexpression of RIOK2 on the reduced migration ability caused by the miR‐4744 mimics treatment (Figure 6B).

### 3.7 Overexpression of RIOK2 could reverse the effects of miR‐4744 on glioma cell invasion

**Figure 7:**
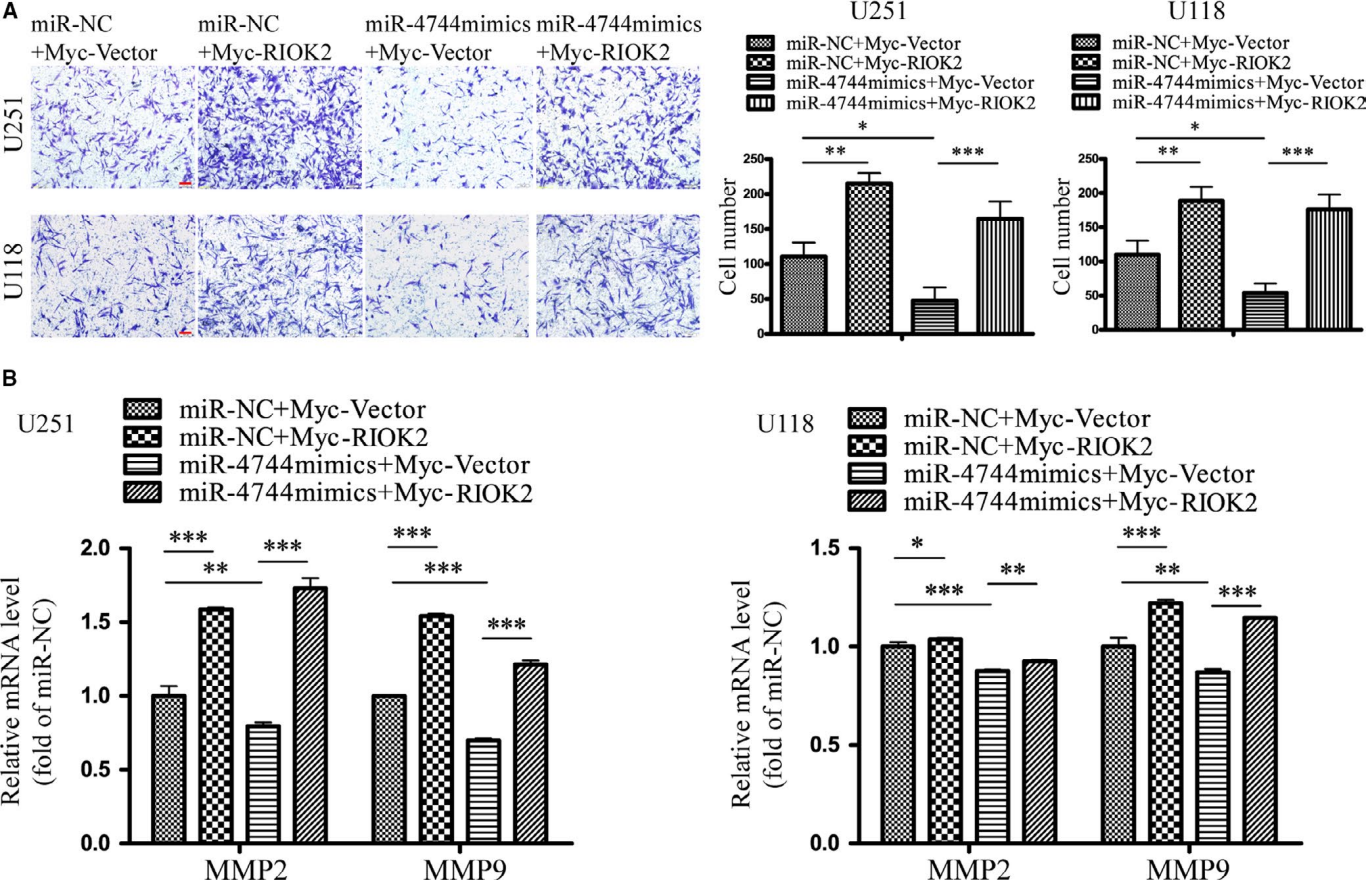
Overexpression of RIOK2 could reverse the effects of miR‐4744 on glioma cell invasion. (A) Transwell invasion assay was performed to assess the reversal effects of RIOK2 overexpression on the decreased cell invasion caused by up‐regulation of miR‐4744 in U251 and U118 cells. Representative images were shown on the left column, and quantitative analyses of the number of cells passing through the Matrigel were shown on the right column. (B) QRT‐PCR was used to evaluate the reversal effects of RIOK2 overexpression on the decreased mRNA levels of MMP2 and MMP9 caused by up‐regulation of miR‐4744 in U251 and U118 cells. Scale bar: 100 μm. *P < .05; **P < .01; ***P < .001

In consistent with our findings in Figure 4B, overexpression of RIOK2 resulted in a significant increase in the invading cell numbers in U251 (*P* = .001) and U118 (*P* = .005) cells (Figure 7A). The miR‐4744 mimics treatment significantly decreased the number of invading cells in U251 (*P* = .020) and U118 (*P* = .033) cells (Figure 7A). Overexpression of RIOK2 partially reversed the decreased cell invasion caused by up‐regulation of miR‐4744 (all *P* < .001, Figure 7A).

QRT‐PCR showed that overexpression of RIOK2 significantly increased the MMP2 and MMP9 mRNA levels in U251 (all *P* < .001) and U118 (MMP2: *P* = .044; MMP9: *P* < .001) cells (Figure 7B). Up‐regulation of miR‐4744 led to a significant decrease in the mRNA levels of MMP2 and MMP9 in U251 (MMP2: *P* = .005; MMP9: *P* < .001) and U118 (MMP2: *P* < .001; MMP9: *P* = .001) cells (Figure 7B). Overexpression of RIOK2 reversed the decrease in MMP2 and MMP9 mRNA levels caused by up‐regulation of miR‐4744 in U251 (all *P* < .001) and U118 (MMP2: *P* = .009, MMP9: *P* < .001) cells (Figure 7B).

### 3.8 Overexpression of RIOK2 could reverse the effect of miR‐4744 on the EMT process in glioma cells

**Figure 8:**
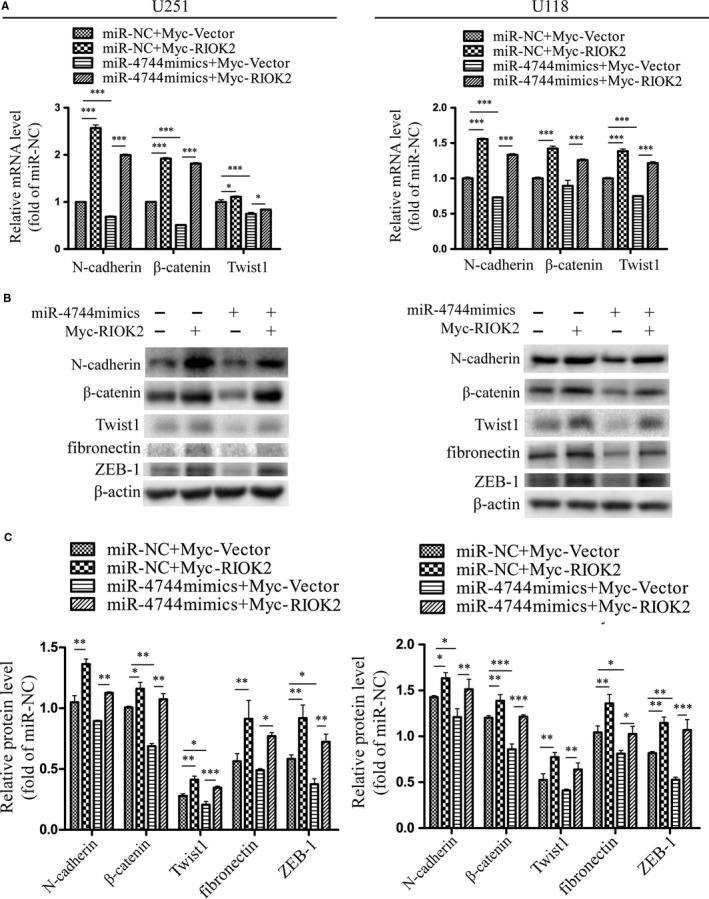
Overexpression of RIOK2 could reverse the effect of miR‐4744 on the EMT process in glioma cells. (A) QRT‐PCR was performed to evaluate the reversal effects of RIOK2 overexpression on the decreased mRNA levels of EMT markers caused by up‐regulation of miR‐4744 in U251 and U118 cells. (B‐C) Western blot was employed to assess the reversal effects of RIOK2 overexpression on the decreased protein levels of the indicated EMT markers caused by up‐regulation of miR‐4744 in U251 and U118 cells. Representative blot images were shown in (B). Quantification graphs were shown in (C). *P < .05; **P < .01; ***P < .001

QRT‐PCR showed that overexpression of RIOK2 resulted in a significant increase in the mRNA levels of mesenchymal markers in U251 (N‐cadherin: *P* < .001; β‐catenin: *P* < .001; Twist1: *P* = .011) and U118 (all *P* < .001) cells, while the miR‐4744 mimics treatment decreased the mRNA expression of mesenchymal markers in U251 (all *P* < .001) and U118 (N‐cadherin: *P* < .001; β‐catenin: *P* = .073; Twist1: *P* < .001) cells (Figure 8A). Overexpression of RIOK2 partially reversed the down‐regulation in the mRNA levels of mesenchymal markers caused by up‐regulation of miR‐4744 in U251 (N‐cadherin: *P* < .001; β‐catenin: *P* < .001; Twist1: *P* = .029) and U118 (all *P* < .001) cells (Figure 8A). Western blot analysis further confirmed the reversal effects of RIOK2 overexpression on the decreased protein levels of N‐cadherin, β‐catenin, Twist1, fibronectin and ZEB‐1 caused by up‐regulation of miR‐4744 in U251 and U118 cells (Figure 8B‐C). Overall, overexpression of RIOK2 could rescue the effects of up‐regulation of miR‐4744 on the migration, invasion and EMT process in glioma cells.

### 3.9 RIOK2 was negatively correlated with miR‐4744 in glioma tissues

**Figure 9:**
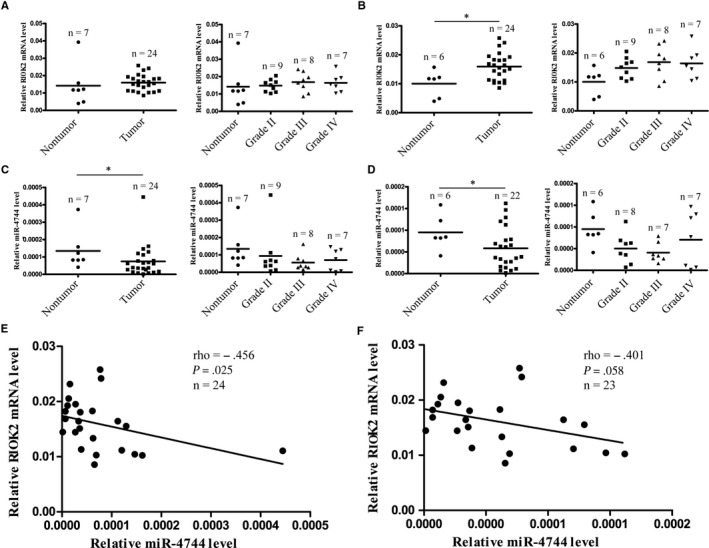
RIOK2‐mRNA is positively correlated with miR‐4744 levels in glioma tissues. QRT‐PCR was applied to measure the RIOK2 and miR‐4744 levels in the pooled glioma group (n = 31), the subgroups with different grades (Grade II, n = 9; Grade III, n = 8; Grade IV, n = 7) and nontumour brain tissues (n = 7). (A‐B) The changes of RIOK2 mRNA level. (C‐D) The changes of miR‐4744 level. (E‐F) Correlation analysis between the miR‐4744 and RIOK2 levels in glioma tissues. (A, C, E) all the subjects were included. (B, D, F) the indicated outliers were excluded from the statistical analysis. *P < .05

**Figure 10:**
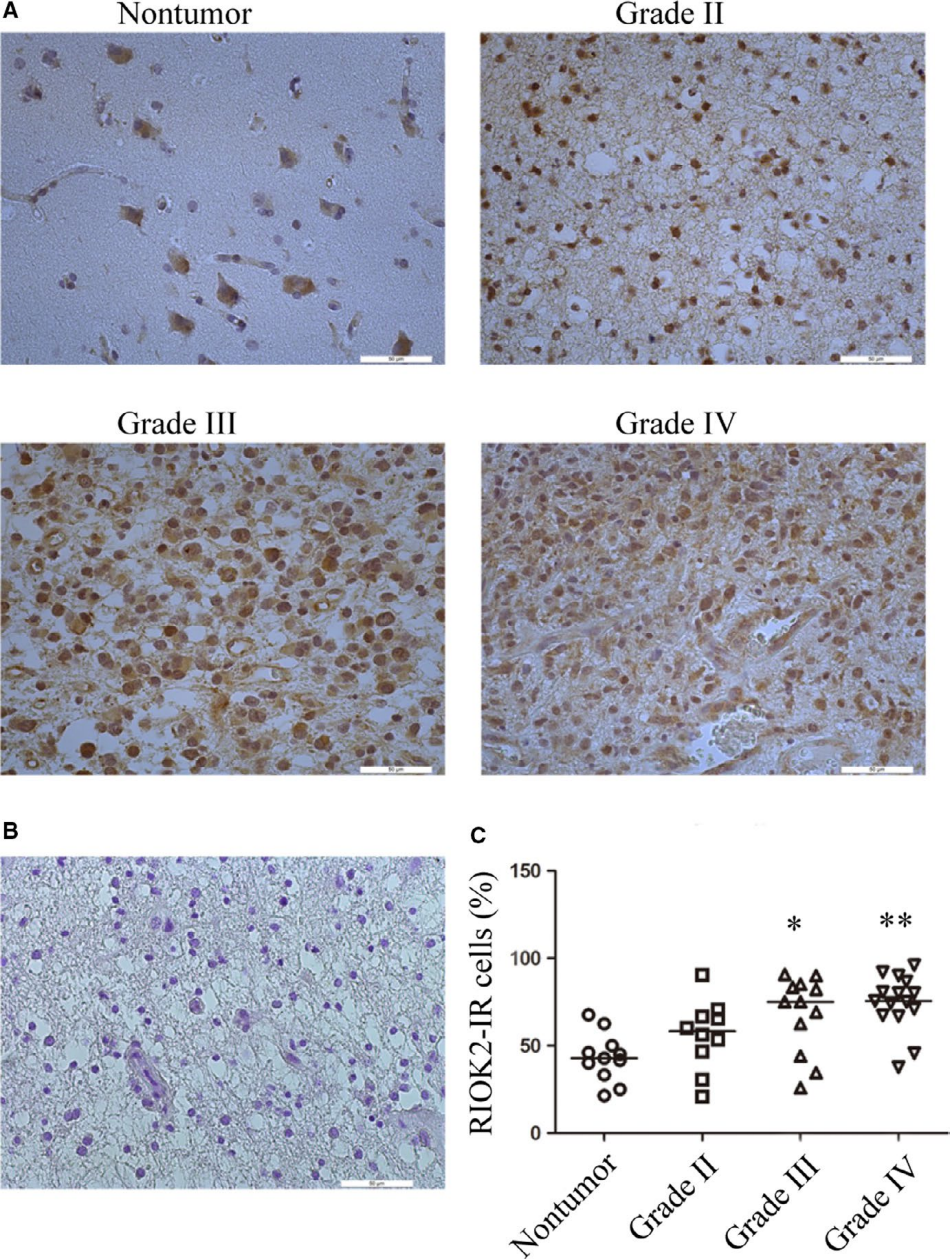
RIOK2 protein level is increased in high‐grade gliomas. Immunohistochemistry was used to detect the immunoreactivity (IR) of RIOK2 in nontumour brain tissues and glioma tissues. (A) Representative images showing the RIOK2‐IR in both cytoplasm and nucleus. Scale bars: 50 μm. (B) Negative control without the primary antibody. (C) Cell counting showed that the percentage of RIOK2‐IR cells was increased in Grade II (n = 10), Grade III (n = 12) and Grade IV (n = 15) glioma tissues as compared to the nontumour brain tissues (n = 11). *P < .05; **P < .01

We employed qRT‐PCR assay to measure the RIOK2 and miR‐4744 levels in 31 clinical specimens, including 7 cases of nontumour brain tissue, 9 cases of Grade II glioma, 8 cases of Grade III glioma and 7 cases of Grade IV glioma. There were no significant differences in the RIOK2‐mRNA or miR‐4744 levels either between the pooled glioma group and the nontumour group or between the various grades of glioma tissues and the nontumour brain tissues (Figure 9A, C). When the outliers were removed (Figure 9B, D), however, we found a significant increase in the RIOK2 mRNA level (*P* = .010) and a significant decrease in the miR‐4744 level (*P* = .025) in the pooled glioma group as compared to the nontumour group. As for the glioma subgroups, the up‐regulation of RIOK2‐mRNA was mostly attributed to Grade III (*P* = .035) and Grade IV groups (*P* = .059), while the down‐regulation of miR‐4744 level was mainly based on Grade II (*P* = .116) and Grade III (*P* = .060) glioma tissues (Figure 9B, D). Moreover, there was a negative correlation between miR‐4744 level and RIOK2 mRNA level in the pooled glioma group (rho = −0.456, *P* = .025, Figure 9E), and the negative correlation still existed when the outlier was removed (rho = −0.401, *P* = .058, Figure 9F). We next used immunohistochemistry followed by cell counting to quantify RIOK2‐IR in 48 paraffin‐embedded specimens (n = 11 for nontumour brain tissues; n = 10 for Grade II; n = 12 for Grade III; and n = 15 for Grade IV). RIOK2‐IR was located in both cytoplasm and nucleus, and it disappeared when the primary antibody was omitted (Figure 10A‐B). Compared with the nontumour group, the percentage of RIOK2‐IR cells was increased in glioma tissues of Grade II (*P* = .076), Grade III (*P* = .015) and Grade IV (*P* = .001) (Figure 10C). These findings revealed up‐regulation of RIOK2 at both mRNA and protein levels and down‐regulation of miR‐4744 level in glioma tissues, especially in high‐grade gliomas.

## 4 DISCUSSION

In this study, we demonstrated that RIOK2 played a promoting role in glioma cell migration and invasion and EMT progression. MiR‐4744 was found to directly bind to the 3'‐UTR of RIOK2 and negatively regulated RIOK2 expression in glioma cells. Overexpression of RIOK2 partially reversed the suppressed effects of miR‐4744 on cell migration, invasion and EMT. In addition, RIOK2 expression was up‐regulated, while miR‐4744 level was down‐regulated in glioma tissues, and a negative correlation was found between them. Collectively, these results revealed upstream and downstream signalling pathways involved in RIOK2‐mediated glioma cell migration and invasion.

The atypical protein kinases, such as RIOK1, RIOK2 and RIOK3, have been studied intensely to understand how they promote ribosomal maturation.^5^ Recently, emerging evidence has suggested an important role of RIO kinases in tumour development and expansion beyond ribosomal biogenesis, including the regulation of cell cycle progression, cell proliferation, migration and invasion. For example, RIOK1 promotes growth and metastasis of colorectal cancer cells in vitro and in vivo.^10^, ^30^ RIOK3 promotes growth, survival, migration and invasion of glioma cells.^9^ RIOK1 and RIOK2 have been shown to drive proliferation and survival in glioblastoma cells.^11^ Here, we reported that RIOK2 contributed to the migration and invasion in glioma cells, which offers a significant supplement for the common functions of RIO kinases in tumour growth and expansion. Further investigations in xenograft animal model are needed to confirm the oncogenic role of RIO kinases in glioma.

MiRNAs affect biological behaviours such as tumour occurrence, proliferation and invasion mostly by regulating target genes. It has been reported that miR‐145 inhibits non‐small‐cell lung cancer growth by targeting RIOK2.^31^ NeuroD2 is post‐transcriptionally targeted by miR‐210, overexpression of NeuroD2 diminishes glioma cell proliferation, migration, and promotes apoptosis under hypoxia.^18^ MiR‐4744 was originally identified by second‐generation sequencing in breast cancer tissues.^20^ We demonstrated that miR‐4744 could bind to the 3’‐UTR of RIOK2 and negatively regulated the RIOK2 expression. More importantly, the inhibitory effects of miR‐4744 on glioma cell migration and invasion were rescued by the overexpression of RIOK2. Therefore, miR‐4744 may inhibit cell migration and invasion by targeting RIOK2, which should be validated in patient‐derived glioma cell lines in the future.

Epithelial‐mesenchymal transition is characterized by loss of epithelial markers (eg E‐cadherin) and gain of mesenchymal markers (eg N‐cadherin, β‐catenin, Twist1), and it has been considered to be an important regulator of the invasiveness of glioma cell.^29^ Here, we found that overexpressing RIOK2 elevated the expression of N‐cadherin, β‐catenin, Twist1, fibronectin and ZEB‐1, whereas the miR‐4744 mimics treatment showed the opposite effects in glioma cells. These findings are in agreement with the promoting role of RIOK2 and the inhibitory effects of miR‐4744 in the migration and invasion of glioma cell. It should be noted that we failed to show the changes of E‐cadherin, probably due to its low expression in glioma cell lines as reported previously.^32^ In addition, the mechanism by which RIOK2/miR‐4744 regulates the EMT process deserves further studies in glioma cells.

In line with the up‐regulation of RIOK2 in lung cancer^33^ and of RIOK3 in glioma tissues,^9^ the elevated expression of RIOK2 was revealed by both qRT‐PCR and immunohistochemical analyses in our glioma specimens, especially in high‐grade gliomas. In addition, RIOK1 was significantly up‐regulated in colorectal cancer and associated with an aggressive and poor survival.^30^ These finding indicated that high expression of RIO kinases might be generalized in different types of cancers and predict poor prognosis. The increase of RIOK2 at both mRNA and protein levels and the inverse correlation between RIOK2‐mRNA and miR‐4744 in glioma tissues provided supporting evidence for the negative regulation of miR‐4744 on its target gene RIOK2 as we demonstrated in vitro. However, the small sample size limited the interpretation of our data, the relationship between miR‐4744 and RIOK2 should be followed up with larger sample sizes.

In summary, knockdown of RIOK2 by siRNAs inhibited the migration, invasion and EMT in glioma cells, while overexpression of RIOK2 showed the opposite effects. Mechanically, RIOK2 was post‐transcriptionally targeted by miR‐4744, overexpression of RIOK2 could reverse the effects of miR‐4744 up‐regulation on the migration, invasion and EMT in glioma cells. The low miR‐4744 and high RIOK2 levels in glioma tissues may contribute to cell migration and invasion through promoting the transition from epithelial cells to mesenchymal phenotype. The present study provides a novel strategy for targeting RIOK2 using small RNAs in the treatments of glioma.

## CONFLICT OF INTEREST

The authors confirm that there are no conflicts of interest.

## AUTHOR CONTRIBUTIONS

SG, YS and RY conceived the study, participated in its design and drafted the manuscript. YS, CL and LJ performed the majority of the experiments and analysed the data. JX and ZS contributed to the Western blot and qRT‐PCR experiments. TZ and DJ collected the clinical samples. All of the authors read and approved the final manuscript.

## Supporting information

Fig S1‐S3

Table S1

Table S2

## Data Availability

The data that support the findings of this study are available from the corresponding author upon reasonable request.
