# Supplementary material for: RIOK2 is negatively regulated by miR‐4744 and promotes glioma cell migration/invasion through epithelial‐mesenchymal transition
Source: J Cell Mol Med. 2020 Mar 3;24(8):4494–509. doi: 10.1111/jcmm.15107 (PMC7176854; doi:10.1111/jcmm.15107)
Supplement: Supplementary file 1 — Fig S1‐S3 [file JCMM-24-4494-s001.docx]

**Supplementary figures and legends:**

**
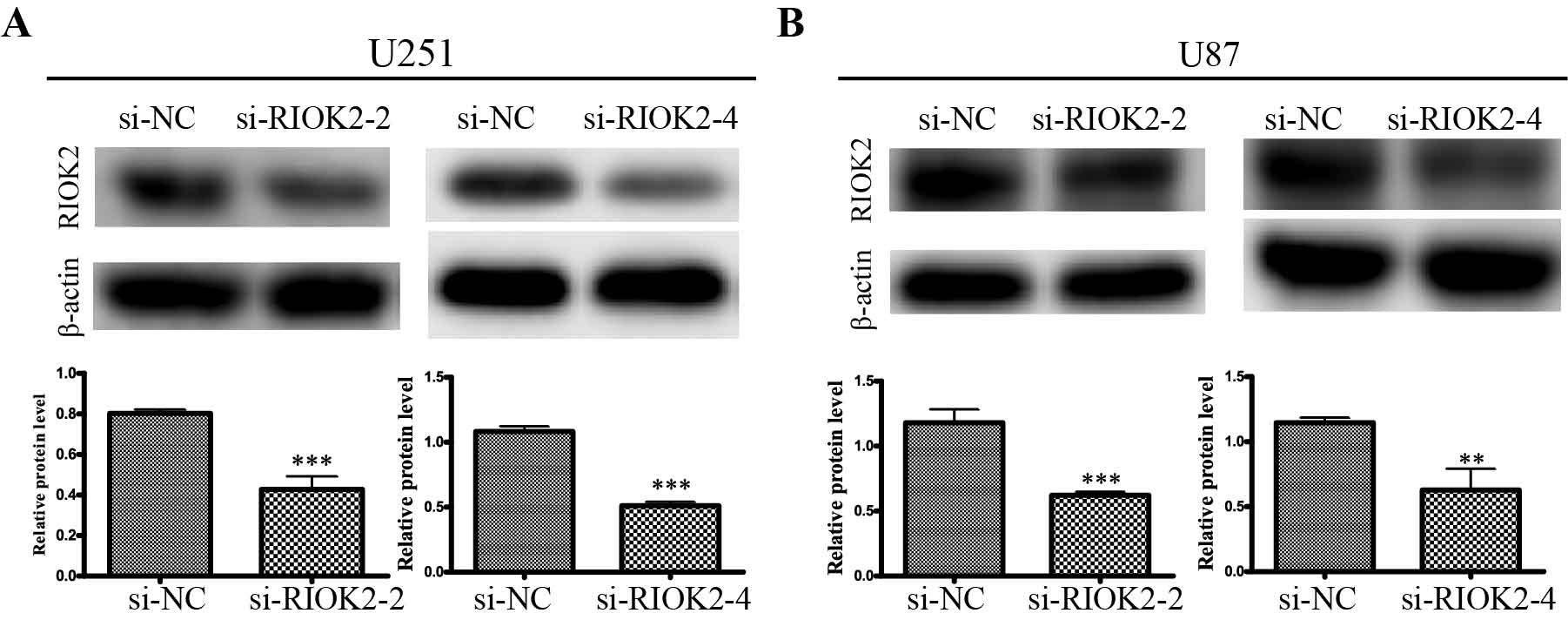
**

**Figure S1 Down-regulation efficiency of RIOK2 siRNAs in glioma cells.** Western blot analysis showed that both siRNAs (siRIOK2-2 and siRIOK2-4) significantly down-regulated the RIOK2 protein levels in U251 and U87 cells. ** *P* < 0.01; *** *P* < 0.001


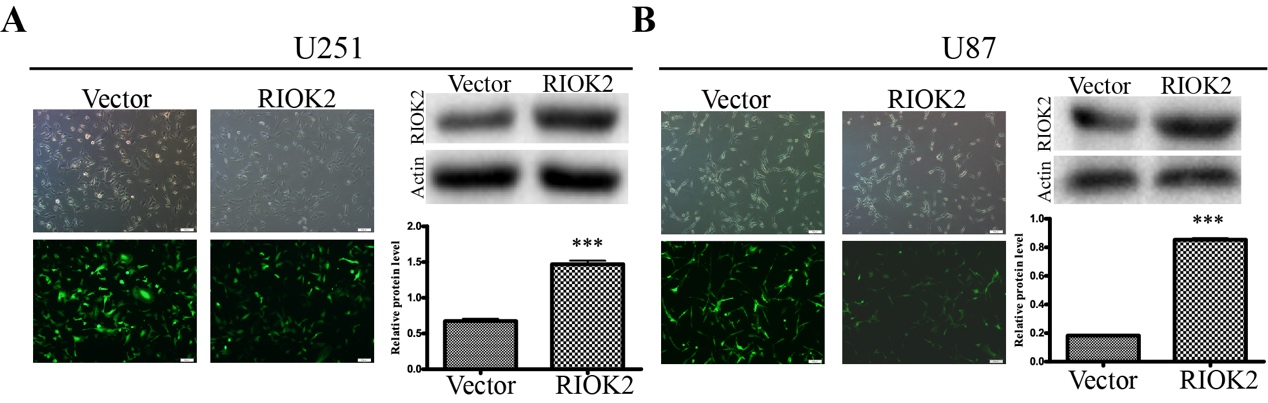


**Figure S2 Overexpression efficiency of RIOK2 in glioma cells.** Combined the GFP and DAPI fluorescence, 90% of cells were infected by lentiviruses produced from the Vector and RIOK2 plasmids. Western blot showed that RIOK2 was abundantly expressed in U251 and U87 cells. Scale bar: 100 μm. *** *P* < 0.001.


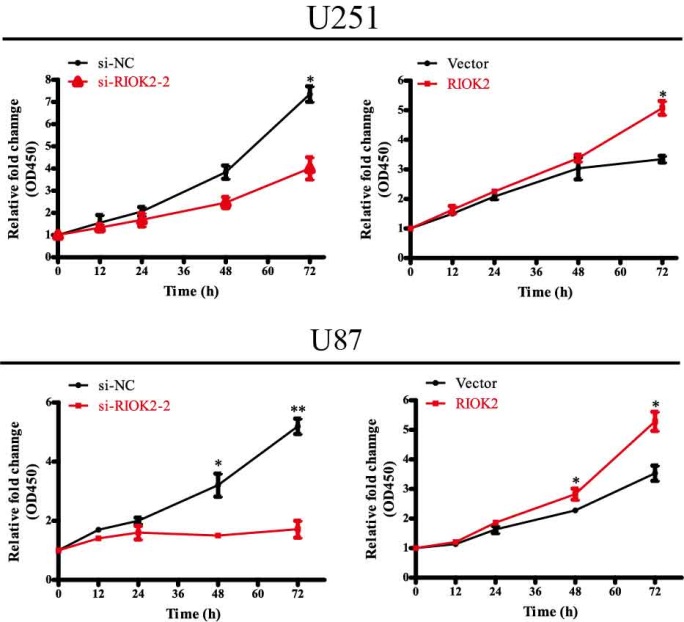


**Figure S3 Effects of RIOK2 down-regulation or overexpression on cell proliferation in glioma cells.** CCK8 assay was used to measure cell viability. In U251 cells, the cell viability was significantly decreased at 72 h after transfection of RIOK2-siRNA, and it was significantly increased in RIOK2-overexpressing cells. In U87 cells, knockdown of RIOK2 caused a significant decrease in the cell viability at 48-72 h, while overexpression of RIOK2 significantly increased the cell viability at 48-72 h. * *P* < 0.05; ** *P* < 0.01.
