# Supplementary material for: RIOK2 is negatively regulated by miR‐4744 and promotes glioma cell migration/invasion through epithelial‐mesenchymal transition
Source: J Cell Mol Med. 2020 Mar 3;24(8):4494–509. doi: 10.1111/jcmm.15107 (PMC7176854; doi:10.1111/jcmm.15107)
Supplement: Supplementary file 2 — Table S1 [file JCMM-24-4494-s002.docx]

**Table S1. Clinico-pathological information for the studied subjects**

| Case No. | Code No. | Gender | Age (years) | Used for | WHO Grade |
| --- | --- | --- | --- | --- | --- |
| 1 | 1233247 | F | 49 | qRT-PCR | Nontumor |
| 2 | 1237928 | M | 53 | qRT-PCR | Nontumor |
| 3 | 1241213 | F | 54 | qRT-PCR | Nontumor |
| 4 | 1250590 | M | 55 | qRT-PCR | Nontumor |
| 5 | 1004728 | M | 63 | qRT-PCR | Nontumor |
| 6 | 1095392 | M | 32 | qRT-PCR | Nontumor |
| 7 | 972078 | F | 49 | qRT-PCR | Nontumor |
| 8 | 1158620 | M | 43 | qRT-PCR | Grade II |
| 9 | 1157139 | M | 42 | qRT-PCR | Grade II |
| 10 | 1161905 | M | 73 | qRT-PCR | Grade II |
| 11 | 1174937 | M | 27 | qRT-PCR | Grade II |
| 12 | 1190502 | M | 31 | qRT-PCR | Grade II |
| 13 | 1196273 | M | 40 | qRT-PCR | Grade II |
| 14 | 1197520 | M | 40 | qRT-PCR | Grade II |
| 15 | 1245545 | M | 27 | qRT-PCR | Grade II |
| 16 | 1236896 | M | 39 | qRT-PCR | Grade II |
| 17 | 1164248 | F | 66 | qRT-PCR | Grade III |
| 18 | 1191197 | M | 68 | qRT-PCR | Grade III |
| 19 | 1234213 | F | 59 | qRT-PCR | Grade III |
| 20 | 1241430 | F | 19 | qRT-PCR | Grade III |
| 21 | 1246522 | M | 34 | qRT-PCR | Grade III |
| 22 | 1260826 | M | 78 | qRT-PCR | Grade III |
| 23 | 1315290 | M | 39 | qRT-PCR | Grade III |
| 24 | 1413682 | F | 44 | qRT-PCR | Grade III |
| 25 | 1204815 | M | 43 | qRT-PCR | Grade IV |
| 26 | 1223892 | M | 66 | qRT-PCR | Grade IV |
| 27 | 1242288 | M | 26 | qRT-PCR | Grade IV |
| 28 | 1252386 | F | 54 | qRT-PCR | Grade IV |
| 29 | 1271138 | M | 30 | qRT-PCR | Grade IV |
| 30 | 1550918 | M | 42 | qRT-PCR | Grade IV |
| 31 | 1204760 | F | 65 | qRT-PCR | Grade IV |

**Note:** F, Female; M, Male; WB, Western blot; qRT-PCR, quantitative real-time PCR; WHO,

World Health Organization
