# Supplementary material for: RIOK2 is negatively regulated by miR‐4744 and promotes glioma cell migration/invasion through epithelial‐mesenchymal transition
Source: J Cell Mol Med. 2020 Mar 3;24(8):4494–509. doi: 10.1111/jcmm.15107 (PMC7176854; doi:10.1111/jcmm.15107)
Supplement: Supplementary file 3 — Table S2 [file JCMM-24-4494-s003.doc]

**Table S2. Primers and siRNAs**

| **Name** | **Primers** | **Sequence(5'-3')** |
| --- | --- | --- |
| MMP2 | Forward | CTGAAGGACACACTAAAGA |
|  | Reverse | CGATGGTATTCTGGTCAA |
| MMP9 | Forward | CATCTTTGTGTCCTACTCTAC |
|  | Reverse | CACCGAAACAGCATTAGC |
| N-cadherin | Forward | CCTCAGTCAACTGCAACCGT |
|  | Reverse | TGGGTCCTGAGCAGTGAATG |
| β-catenin | Forward | CCAGCCGACACCAAGAAG |
|  | Reverse | CGAATCAATCCAACAGTAGCC |
| Twist 1 | Forward | TCGGACAAGCTGAGCAAGAT |
|  | Reverse | TCCATCCTCCAGACCGAGAA |
| RIOK2 | Forward | ACATGAGCCGAGATGACTTC |
|  | Reverse | AACCGATAGCCCTGGACA |
| β-actin | Forward | CCAACCGCGAGAAGATGA |
|  | Reverse | CCAGAGGCGTACAGGGATAG |
| U6 | Forward | AACGCTTCACGAATTTGCGT |
|  | Reverse | CTCGCTTCGGCAGCACA |
| si-NC | Sense | GCUCGCCUGUCUACUAACUAAdTdT |
|  | Antisense | UUAGUUAGUAGACAGGCGAGCdTdT |
| si-RIOK2-2 | Sense | GUCCAGGGCUAUCGGUUGAdTdT |
|  | Antisense | UCAACCGAUAGCCCUGGACdTdT |
| si-RIOK2-4 | Sense | GAACGGAACUGUCUAGAAGdTdT |
|  | Antisense | CUUCUAGACAGUUCCGUUCdTdT |
